# Lifestyle Factors, Medication Use and Risk for Ischaemic Heart Disease Hospitalisation: A Longitudinal Population-Based Study

**DOI:** 10.1371/journal.pone.0077833

**Published:** 2013-10-16

**Authors:** Anthony S. Gunnell, Kristjana Einarsdóttir, Daniel A. Galvão, Sarah Joyce, Stephania Tomlin, Vicki Graham, Caroline McIntyre, Robert U. Newton, Tom Briffa

**Affiliations:** 1 Edith Cowan University Health and Wellness Institute, Edith Cowan University, Joondalup, Western Australia, Australia; 2 Edith Cowan University Survey Research Centre, Edith Cowan University, Joondalup, Western Australia, Australia; 3 Telethon Institute for Child Health Research, Centre for Child Health Research, University of Western Australia, Subiaco, Western Australia, Australia; 4 Epidemiology Branch, Western Australian Department of Health, East Perth, Western Australia, Australia; 5 School of Population Health, University of Western Australia, Crawley, Western Australia, Australia; University of Louisville, United States of America

## Abstract

**Background:**

Lifestyle factors have been implicated in ischaemic heart disease (IHD) development however a limited number of longitudinal studies report results stratified by cardio-protective medication use.

**Purpose:**

This study investigated the influence of self-reported lifestyle factors on hospitalisation for IHD, stratified by blood pressure and/or lipid-lowering therapy.

**Methods:**

A population-based cohort of 14,890 participants aged 45+ years and IHD-free was identified from the Western Australian Health and wellbeing Surveillance System (2004 to 2010 inclusive), and linked with hospital administrative data. Adjusted hazard ratios for future IHD-hospitalisation were estimated using Cox regression.

**Results:**

Current smokers remained at higher risk for IHD-hospitalisation (adjusted HR=1.57; 95% CI: 1.22-2.03) after adjustment for medication use, as did those considered overweight (BMI=25-29 kg/m^2^; adjusted HR=1.28; 95% CI: 1.04-1.57) or obese (BMI of ≥30kg/m^2^; adjusted HR=1.31; 95% CI: 1.03-1.66). Weekly leisure-time physical activity (LTPA) of 150 minutes or more and daily intake of 3 or more fruit/vegetable servings reduced risk by 21% (95% CI: 0.64-0.97) and 26% (95% CI: 0.58-0.96) respectively. Benefits of LTPA appeared greatest in those on blood pressure lowering medication (adjusted HR=0.50; 95% CI: 0.31-0.82 [for LTPA<150 mins], adjusted HR=0.64; 95% CI: 0.42-0.96 [for LTPA>=150 mins]). IHD risk in smokers was most pronounced in those taking neither medication (adjusted HR=2.00; 95% CI: 1.41-2.83).

**Conclusion:**

This study confirms the contribution of previously reported lifestyle factors towards IHD hospitalisation, even after adjustment for antihypertensive and lipid-lowering medication use. Medication stratified results suggest that IHD risks related to LTPA and smoking may differ according to medication use.

## Introduction

Identification and management of lifestyle risk factors for cardiovascular disease (CVD), together with advances in preventive pharmacotherapy have reduced CVD-related mortality in most high-income countries [1], including Australia [2]. Despite this promising trend, death from CVD (of which ischaemic heart disease [IHD] is the most common form) mostly leads national statistics in these jurisdictions [1]. IHD remains the leading underlying cause of death in Western Australia, with an age-standardised death rate of 79.5 deaths per 100,000 people [3]. This is almost twice the rate of the 2^nd^ highest cause of death (malignant neoplasms of the digestive organs) which has a standardised death rate of 44 per 100,000 [3]. 

A number of biomedical and behavioural risk factors have been identified for the development of CVD. These risk factors include smoking, diabetes mellitus (DM), insufficient physical activity, hypertension, high cholesterol, overweight and obesity, and an unhealthy diet [4,5]. Current guidelines for people with hypertension and/or dyslipidaemia include pharmacologic and lifestyle interventions for their control and management [6,7]. The effectiveness of such medications in preventing premature IHD is well documented [7]; however an individual’s response to pharmacotherapy is variable and influenced by such factors as genetics, gender, age, weight, smoking status and physical activity levels [8]. We investigated the risks associated with various lifestyle factors, not only in overall terms but also within groups of people on specific blood pressure and lipid-lowering medications. 

## Methods

### Ethics Statement

The study was approved by the Human Research Ethics Committees of Edith Cowan University and the Western Australian Department of Health, and has therefore been performed in accordance with the ethical standards laid down in the 1964 Declaration of Helsinki and its later amendments. 

This population-based cohort study utilised self-reported lifestyle survey information (from the Health and Wellbeing Surveillance System [HWSS]) individually linked with hospital admission data (both held by the Western Australian Department of Health). The HWSS is a comprehensive monthly survey commissioned by the Health Department to provide information on a wide range of issues pertaining to the Western Australian population’s physical and mental well-being. It utilises computer-assisted telephone interviewing to assess approximately 6,000 Western Australians each year who are selected from the WA White Pages^®^ telephone directory using a stratified random process with over-sampling representative to the population in rural and remote areas. Each year since its inception, more than 75% of those contacted completed the survey [9] and a majority (77% in 2010) of participants provided their name, address and date of birth for the purpose of linkage with administrative health data. Only those HWSS participants who provided consent for their information to be used in this manner were linked to other registries for this study. The probabilistic matching procedures used to link individuals are based on full name and address, phonetic compression algorithms and other identifiers, and have been estimated to be 99.89% accurate [10]. This linkage allowed identification of incident IHD hospitalisations following an initial survey date and provided information on medication use, behavioural factors and demographics at the time of survey.

### Study Population

Between 1 January 2004 and 31 December 2010, some 40,445 surveys for which consent was provided for data linkage, were completed as part of the HWSS. Where participants had been surveyed more than once (1,671 people) during the study period, their first survey was included for analysis. Upon further restriction to those eligible for a subsidised health check-up with their doctor, i.e. aged 45+ years and without prior IHD-hospitalisation, a final study cohort of 14,890 remained.

### Study Variables

Exposures of interest included smoking status (never more than 100 cigarettes, ex-smoker, current-smoker), leisure-time physical activity (LTPA) in past week (none, <150min, ≥150min), fruit and/or vegetable intake daily (<3, ≥3 pieces), and body mass index (BMI; <25, 25-29, 30+ kg/m^2^). These exposures were also stratified by self-reported current use (at time of survey) of medications for high blood pressure or elevated total cholesterol. Participants were grouped into 4 categories; no current use of antihypertensive or lipid-lowering medications, antihypertensive medications only, lipid-lowering medications only, both antihypertensive and lipid-lowering medications. The primary outcome was hospitalisation for IHD according to the principal diagnosis (International Classification of Diseases [ICD] – version 9 [ICD-9] 410-414 or ICD-10 I20-I25) post survey date. The study population was restricted to those having no previous IHD-related hospitalisation (in any of the 22 administrative diagnostic categories) within 5 years of the survey date.

LTPA included minutes of walking (in at least 10 min blocks), moderate and vigorous activity, where minutes of vigorous activity were multiplied by two (as described in the Australian physical activity guidelines [11]). For those aged 65+, no weighting was applied for vigorous activity in order to improve comparability between years due to the question not being asked for that age group prior to 2008. Few respondents aged 65+ reported being vigorously active from 2008 onwards. Minutes of activity were grouped as meeting public health guidelines for sufficient levels of LTPA (0, <150 minutes, ≥150 minutes). For those whose total LTPA per week exceeded 1,680 minutes, their summed value was re-coded to 1,680 minutes for analysis (as recommended by the Australian Institute of Health and Welfare [11]). 

Categorisation of self-reported daily fruit and vegetable intake was based on evidence suggestive of a protective effect of 3 or more fruit/vegetables daily on fatal-IHD risk [12]. BMI categories, also self-reported, were classified as underweight/normal (<25 kg/m^2^), overweight (25-29 kg/m^2^) or obese (30+ kg/m^2^) [13]. Sedentary behaviour was categorised according to the mean and quartiles for time in this state. These sedentary categories (<10, 10-14, 15-21, 22+ hours per week) were based on participant recall of the average number of hours per week spent watching TV, videos, or using a computer for any reason. Prior to 2006, these responses for participants younger than 65 years did not exclude work time whereas from 2006 work time sedentary behaviour was excluded.

In order to determine participants’ medication usage at time of survey, participants were asked if they took any medication for blood pressure, and separately asked if they took medication for high cholesterol. Disaggregation to class of anti-hypertensive or lipid-lowering agent was not possible, nor was ascertainment of dosage or adherence.

Additional confounding factors adjusted for in the analyses were age at survey (45-59, 60-70, 70+ years), year of survey, gender, diagnosis of DM in any hospitalisation diagnostic category within 5 years prior to the survey date, and Charlson comorbidity index score (based on a modified Dartmouth-Manitoba algorithm [14]) within five years prior to the survey date. Charlson comorbidity index and prior DM were generated using ICD-9 and ICD-10AM codes contained within the diagnostic hospital records. 

### Statistical Analyses

Person-time was calculated from the date of survey until the admission date for a principal IHD-hospitalisation, death, or until end of follow-up (31 Dec 2010), whichever occurred first. Cox proportional hazards regression models were used to estimate hazard ratios (HR) with 95% confidence intervals (CIs) for future IHD-hospitalisation. The final adjusted model included age, sex, smoking status, Charlson comorbidity index, LTPA, sedentary behaviour, BMI, daily fruit and vegetable intake, survey year, and known DM. Socioeconomic index for areas (SEIFA) and region (metro or regional residence) were not included in the adjusted model as they were not significantly associated with the outcome in crude Cox models.

The proportional hazards assumption that the ratio of mortality rates according to the exposure variable remained constant for the adjusted models was tested by inclusion of an interaction term with ln(1 + survival time) in the model. This identified one violation of the proportional hazards, namely BMI. We further tested this by estimating HRs and their confidence intervals separately by number of years of follow-up. A 24-27% higher HR estimate (for 25-29 and 30+ kg/m^2^ respectively) was observed in the 1^st^ year of follow-up, compared to the 2^nd^ follow-up period (2 years), however overlapping confidence intervals suggest the differences were not significant. Subsequent HR estimates for longer follow-up periods differed by about 3% to their previous year’s HR estimate and similarly had overlapping confidence interval compared with the 1^st^ year confidence intervals. We therefore presented the HR estimates for the entire period rather than stratified by year. To test whether or not the HR estimates for other risk factors might differ as a result of any possible violation in propotional hazards by BMI, an adjusted Cox regression model stratified by BMI (BMI was placed in a strata statement) was performed. Estimates did not vary by more than 0.05 following stratification of BMI. 

For all analyses, a two sided P<0.05 was considered statistically significant. The statistical software SAS 9.3 was used to perform all analyses.

## Results

### Baseline Characteristics

Females made up 59% (n=8,776) of the final study cohort (Table 1) which ranged in age from 45 to 97 years. Fifty eight percent of those surveyed reported no current use of antihypertensive or lipid-lowering medications, just over half of those being aged between 45 and 60 years. Self-reported current use of antihypertensive medications (with or without lipid-lowering medications) increased with age; however the use of ‘lipid-lowering medications only’ showed relative stability over all age groups. This latter exposure category also possessed a slightly higher proportion of males (45%) compared with the other three medication categories. Prior hospitalisation for DM was more prevalent in those reporting use of either medication (compared to those reporting none), and DM was most prevalent in the combined medication group where 16% of participants had a DM-hospitalisation within 5 years prior to the survey date. Sufficient LTPA was more common in people who did not take medications, whereas sedentary behaviour, DM and high BMI were more common in people who took medications. Being a current smoker was more common in people who were not on any medications, while being a former smoker and eating more fruit and/or vegetables was more common for people who were currently taking medications.

**Table 1:** Study population characteristics by exposure category: Persons aged 45+ years with no prior^^^ IHD, 2004-2010.

	No medications (n=8640)	Hypertension medications (n=3081)	Lipid-lowering medications (n=1336)	Both medications (n=1833)
	n (%)	n (%)	n (%)	n (%)
Male	3551 (41.1)	1224 (39.7)	605 (45.3)	734 (40.0)
Age at Survey (years)				
45-59	4639 (53.7)	811 (26.3)	439 (32.9)	355 (19.4)
60-70	2141 (24.8)	963 (32.3)	468 (35.0)	624 (34.0)
70+	1860 (21.5)	1307 (42.4)	429 (32.1)	854 (46.6)
Leisure-Time Physical Activity				
None	1913 (22.1)	849 (27.6)	321 (24.0)	556 (30.3)
< 150min (inc walking)	2243 (26.0)	887 (28.8)	363 (27.2)	543 (29.6)
≥150min (inc walking)	4484 (51.9)	1345 (43.6)	652 (48.8)	734 (40.0)
Weekly Sedentary Activity (sitting)				
<10hr	2103 (24.3)	539 (17.5)	249 (18.6)	280 (15.3)
10-14hr	2433 (28.2)	801 (26.0)	323 (24.2)	423 (23.1)
15-21	2240 (25.9)	849 (27.6)	361 (27.0)	505 (27.6)
22+	1864 (21.6)	892 (29.0)	403 (30.2)	625 (34.1)
Smoking Status				
Never	4027 (46.6)	1563 (50.7)	612 (45.8)	875 (47.7)
Ex-smoker	3274 (37.9)	1209 (39.2)	552 (41.3)	776 (42.3)
Current	1339 (15.5)	309 (10.0)	172 (12.9)	182 (9.9)
3 or more fruit or veg daily (yes)*	7714 (89.3)	2810 (91.2)	1225 (91.7)	1647 (89.8)
BMI (kg/m^2^)**				
< 25	3594 (41.6)	883 (28.7)	441 (33.0)	453 (24.7)
25 - 29	3077 (35.6)	1078 (35.0)	534 (40.0)	724 (39.5)
30+	1545 (17.9)	955 (31.0)	298 (22.3)	586 (32.0)
Previous Diabetes Mellitus (yes)^	252 (2.9)	242 (7.8)	112 (8.4)	298 (16.3)
Person-Years of follow up (mean)	3.2	3.0	2.9	2.8
Person-Years of follow up (total)	27,353.8	9,303.6	3,881.6	5,091.1
IHD-hospitalisations (n)~	255 (3.0)	132 (4.3)	62 (4.6)	107 (5.8)
Crude IHD-hospitalisation rate (per 100,000 PY)	932.2	1418.8	1597.3	2101.7

* Missing values recoded to zero for 65 missing responses to fruit and/or vegetables, and 163 missing responses for mins of leisure-time physical activity.

** Excluded missing values for BMI (n=722).

^ Separation date within 5 years prior HWSS date.

~ Principal diagnosis of IHD at admission.

The mean number of person-years of follow-up ranged from 2.8 to 3.2 years between exposure groups, with a maximum of almost seven years for each of the four exposure categories. The crude IHD-hospitalisation rate ranged from 932 per 100,000 person-years in the ‘no medication’ category, to 2,102 IHD-hospitalisations per 100,000 person-years in the combined medication group.

### Future IHD-hospitalisation, lifestyle factors and current medication use

After adjustment for current medication use, all *a priori* selected risk factors aside from sedentary behaviour demonstrated significant increased or decreased risk for future IHD-hospitalisation (Table 2), regardless of medication use. Weekly LTPA of 150 minutes or more was associated with a 21% (adjusted HR=0.79; 95% CI: 0.64-0.97) decreased risk, and 3 or more daily servings of fruit/vegetables translated to a 26% (adjusted HR=0.74; 95% CI: 0.58-0.96) decreased risk for IHD-hospitalisation. Conversely, overweight (BMI=25-29kg/m^2^) or obesity (BMI of ≥30kg/m^2^) afforded an increased risk of 28% (adjusted HR=1.28; 95% CI: 1.04-1.57) and 31% (adjusted HR=1.31; 95% CI: 1.03-1.66) respectively, and current smoking provided a corresponding 57% (adjusted HR=1.57; 95% CI: 1.22-2.03) increased risk for future IHD-hospitalisation. Addition of interaction terms to the Cox regression model revealed no significant interaction effects between the aforementioned risk factors and medication use in future IHD-hospitalisation.

**Table 2:** Hazard Ratios for future IHD-hospitalisation by self-reported medication use and lifestyle risk factors.

	Hazard Ratios (95% CIs) ^#^				
	No medications	Hypertension medications	Lipid-lowering medications	Both medications	Total
Total (n)*	8216	2916	1273	1763	14168
Event (n)*	251	126	61	100	538
Weekly Leisure-Time Physical Activity					
None	Ref	Ref	Ref	Ref	Ref
< 150min	0.93 (0.66-1.30)	0.50 (0.31-0.82)	1.02 (0.50-2.04)	0.77 (0.46-1.29)	0.80 (0.64-1.00)
≥150min	0.84 (0.62-1.14)	0.64 (0.42-0.96)	0.81 (0.42-1.59)	0.86 (0.54-1.39)	0.79 (0.64-0.97)
BMI (kg/m^2^)					
< 25	Ref	Ref	Ref	Ref	Ref
25 - 29	1.39 (1.05-1.84)	1.09 (0.70-1.71)	0.80 (0.44-1.48)	1.67 (0.94-2.94)	1.28 (1.04-1.57)
30+	1.23 (0.85-1.77)	1.19 (0.74-1.91)	1.54 (0.78-3.08)	1.50 (0.82-2.76)	1.31 (1.03-1.66)
Smoking Status					
Never	Ref	Ref	Ref	Ref	Ref
Ex-smoker	1.14 (0.85-1.53)	1.14 (0.77-1.70)	0.93 (0.52-1.65)	1.25 (0.80-1.95)	1.12 (0.93-1.36)
Current	2.00 (1.41-2.83)	1.48 (0.84-2.62)	1.47 (0.66-3.26)	0.86 (0.40-1.84)	1.57 (1.22-2.03)
Daily Fruit or Vegetable intake					
<3 servings	Ref	Ref	Ref	Ref	Ref
≥3 servings	0.76 (0.53-1.09)	0.70 (0.40-1.20)	0.54 (0.25-1.17)	0.89 (0.45-1.75)	0.74 (0.58-0.96)
Weekly Sedentary Activity					
<10hr	Ref	Ref	Ref	Ref	Ref
10-14hr	0.91 (0.63-1.31)	1.15 (0.66-2.01)	0.87 (0.37-2.04)	0.88 (0.47-1.67)	0.95 (0.73-1.23)
15-21	1.10 (0.77-1.56)	1.20 (0.69-2.07)	1.18 (0.54-2.59)	0.85 (0.46-1.58)	1.09 (0.83-1.38)
22+	1.08 (0.75-1.56)	0.83 (0.47-1.47)	1.26 (0.58-2.75)	0.94 (0.52-1.71)	1.01 (0.78-1.30)

* Numbers differ to Table 1 due to exclusion of any missing values in the Cox regression analyses.

^#^ Cox model includes age, sex, smoking status, Charlson index, leisure-time physical activity, sedentary activity level, body mass index, daily fruit and vegetable intake, survey year, diabetes hospitalisation (within 5 yrs prior survey date).

When adjusted hazard ratios were estimated for risk factors within sub-groups of medication users (none, antihypertensive medications only, lipid-lowering medications only, concurrent antihypertensive and lipid-lowering medications) the beneficial effects of LTPA were only observed in those on antihypertensive medications only. However within this antihypertensive medication sub-group, significant protective effects of 50% (adjusted HR=0.50; 95% CI: 0.31-0.82) and 36% (adjusted HR=0.64; 95% CI: 0.42-0.96) were observed for <150 minutes and ≥150 minutes respectively. Within the ‘no medication’ sub-group, current smoking doubled the risk for IHD-hospitalisation (adjusted HR=2.00; 95% CI: 1.41-2.83) and a BMI of 25-29kg/m^2^ was associated with a 39% (adjusted HR=1.39; 95% CI: 1.05-1.84) increased risk, compared to those with a BMI of less than 25kg/m^2^. Neither smoking nor BMI demonstrated any significant increased risks within other sub-groups of medication use, although in some cases this is likely due a lack of sufficient power within the medication sub-groups.

## Discussion

In this population-based cohort study of 14,890 Western Australians, a significant increased risk (adjusted for commonly reported risk factors) of future IHD-hospitalisation was observed for those who currently smoked (57% higher), or were overweight (28% higher) or obese (31% higher). In contrast, meeting public health guidelines of 150 minutes of physical activity and daily intake of 3 or more servings of fruit or vegetables appeared to decrease risk by 21% and 26%, respectively.

Medication sub-group analyses suggested that benefits or risks associated with certain lifestyle factors might be more or less pronounced, depending upon medication use. The benefits from not being a current smoker were greatest in individuals who were not taking antihypertensive and/or lipid-lowering medications, and benefits of higher levels of physical activity appeared most pronounced in the antihypertensive medication group. 

Estimates of gain from pharmacological therapies in terms of CVD risk have ranged from 20% to 30% [15,16]. This is likely due to differences in medication efficacy associated with age, sex and genetic characteristics, in addition to a number of lifestyle factors for IHD which may have causal roles individually or in combination with others [17]. The increased risk of smoking on IHD has been published widely [2,18–20]. Overall increased risk (an adjusted odds ratio of 2.87) for myocardial infarction as a function of number of cigarettes in current smokers (up to 9 times higher risk in those smoking >40 cigarettes per day) compared to that of non-smokers has been reported from the INTERHEART study [5]. Whilst our 57% increased risk was relatively low in comparison, the broader outcome utilised in our study (IHD) probably explains the lower risk. Smoking did however confer the greatest increase in risk for IHD-hospitalisation in our study (after antihypertensive and lipid-lowering medication use) and interestingly, results suggested the risk might be attenuated somewhat by the use of antihypertensive and/or lipid-lowering medications. Synergistic effects between smoking and hypertension in CVD incidence have been reported previously [21]. Normalisation of blood pressure through medication use in current smokers could therefore explain the drop in risk to non-significant levels in our study. Interestingly, the benefits of statin use (for controlling dyslipidaemia) on IHD risk in smokers have also been evidenced previously including a review of the landmark statin trials [22]. The authors concluded that (in relation to IHD primary prevention) “One consistent finding across both primary prevention statin trials was that the lowest risk of events in any group was in the nonsmokers on active treatment. The highest risk of events in any group was in the smokers on placebo.” [22]. Given these previous findings, it is perhaps not surprising that the lowest risk for IHD-hospitalisation in current smokers was clearly held by those participants on both anti-hypertensive and lipid-lowering medications and those at highest risk were smokers on neither medication type. 

Obesity has consistently been shown to contribute to risk for IHD in large, well designed studies [23–25]. In the absence of waist-to-hip ratio measurements, which may be a more accurate measure in terms of associated risk for obesity and IHD [25], BMI continues to be useful as a method of adjustment for confounding which may itself be causal [26]. Yusuf et al [25] reported a 44% increased risk (top BMI quintile versus the bottom quintile) for acute myocardial infarction in a large (12,461 cases) case-control study. The slightly attenuated risks observed in our study (up to 31% increased risk for obese participants) are likely due to the broader outcome measure used in our study (IHD rather than myocardial infarction). A recent Danish study [26], which included 11,056 IHD events, found a 26% increased risk of IHD for every 4 kg/m^2^ increase in BMI. This suggests our observed risks for overweight and obese participants are comparably low, however the Danish study did not adjust for insufficient physical activity or fruit/vegetable intake which may have contributed toward the relatively high observed risk.

The impact of physical activity on IHD incidence and mortality has been reported previously [4,17,19,27,28]. Among the range of benefits attributable to physical activity are its effects on glucose control in individuals with type-2 DM, reduced waist circumference, improvements in high-density lipoprotein-cholesterol (HDL-C), reduced total and visceral fat, and decreased blood pressure [28]. Evidence also suggests that physical activity may play a role in blood pressure control independently of, and in combination with, the use of antihypertensive medications. In a study by Calhoun et al [29], reductions in systolic and diastolic blood pressure were achieved following a 16 week aerobic exercise regimen undertaken by a group of men with severe hypertension who were receiving up to three antihypertensive agents. Reductions in diastolic blood pressure remained after 32 weeks of exercise, even following withdrawal of some antihypertensive medications [29]. In line with this evidence, our study suggested a reduction in risk for IHD-hospitalisation could be attained through increased physical activity even after taking into account participants’ use of cardio-protective medications. In fact, the largest benefits of physical activity were observed within the participant group actually taking blood pressure lowering medications only. 

Sufficient fruit and vegetable intake has been implicated as a risk factor of both fatal and non-fatal IHD [5,12,30–32]. Our finding of a 26% reduced risk for IHD in those consuming three or more daily servings of fruit and/or vegetables, reflects results from a prospective study of 44,561 men and women in the UK where vegetarians had a 32% lower risk of IHD than non-vegetarians [31]. Authors of the UK study suggested much of the reduced risk might be explained by the effects of a vegetarian lifestyle on IHD risk factors such as non-HDL cholesterol levels and systolic blood pressure [31]. In our study, a protective association with daily fruit/vegetable intake remained even after adjustment for lipid and blood pressure lowering medications, and many other IHD risk factors.

The population-based nature of this study means that results can be generalised to the broader community who have access to a listed telephone line. Whilst there are areas (particularly remote to very-remote areas) in Western Australia with limited or no access to telephones, the proportion of the overall population is very low. However it should be noted that this inaccessible population is likely to contain a higher proportion of indigenous people than is represented by the study cohort. Importantly, since data were available for all hospitals within Western Australia, we are likely to have included the vast majority of those who develop IHD as very few are treated in the community [33] and migration from Western Australia is relatively low, particularly in older age groups [34]. 

Although primary exposures (medication use) and secondary lifestyle exposures were self-reported, this is unlikely to lead to a differential misclassification of the exposures due to the prospective nature of the study. It is therefore most likely that any misclassification of exposure would underestimate the actual risk estimates. Our inability to identify which antihypertensive or lipid-lowering medication(s) participants are taking, the dosage, and adherence are limitations of this study. General Practitioner prescription estimates from the BEACH report [35] suggest that around 80% of antihypertensive medications are likely to be ACE inhibitors or angiotensin II agonists (plain or combination medications). Lipid-lowering medications will likely include one of the three commonly prescribed statins (Atorvastatin, Rosuvastatin or Simvastatin). Adherence to cardiovascular medications in a sample of Australians has been estimated previously at around 67% [36]. Also, adherence to medication is likely to be associated to a degree with healthy lifestyle factors overall [37]. It is possible therefore, that the decreased risk for IHD-hospitalisation observed in those on antihypertensive medication who have increased physical activity (in the medication-stratified analyses) is in part due to greater adherence to medication. However, the lack of a beneficial effect related to being physically active in the other medication groups suggests adherence is not the sole reason for this finding. The availability of only a single observation time point for exposure measurement may provide some inaccuracies in terms of exposure classification since the behaviour of participants may change over time. This is likely to be more prominent for participants contributing large amount of person-time than those with less, due to the likelihood that time-points closer to the survey date will more accurately reflect the participants’ actual lifestyle factors. Whilst this may increase misclassification of the exposure, the similarities between mean person-time contributions for the four medication exposure groups should lessen any differential bias. It is reassuring that the patterns illustrate increased risks associated with smoking and BMI, and decreased risks associated with physical activity and vegetable/fruit intake on future IHD hospitalisation. These are both highly plausible and have been evidenced previously in other studies [1,5,17,38,39]. Misclassification of the outcome is unlikely since diagnoses are derived from administrative hospital data where diagnoses have been made by hospital clinicians. The sample size for this study was relatively large and as such had adequate power to detect significant effects related to the primary exposure and the lifestyle factors prior to stratification by medication type. However, the sample size is limited with respect to the aforementioned medication-stratified analyses. As such, the absence of significant findings for some lifestyle factors in stratified analyses should be interpreted with caution.

In conclusion, our study highlights the contribution of smoking, overweight and obesity, insufficient physical activity, and low daily fruit and vegetable intake towards IHD hospitalisation, even after adjustment for antihypertensive and lipid-lowering medication use, in the Western Australian population. It confirms the importance of prevention and management of such modifiable risk factors at a population level to bring about reductions in IHD incidence and mortality in the community. Furthermore, given the potential medication-stratified differences in risk attributable to lifestyle factors identified in this study, further investigation of possible interactive effects may be warranted.
